# Alteplase and Angioedema: Can Clinical Exome Sequencing Redefine the Paradigm?

**DOI:** 10.3390/life16020200

**Published:** 2026-01-26

**Authors:** Marina Tarsitano, Maurizio Russo, Vincenzo Andreone, Maria Bova, Francesco Palestra, Paolo Candelaresi, Giovanna Servillo, Anne Lise Ferrara, Gilda Varricchi, Luigi Ferrara, Stefania Loffredo, Massimiliano Chetta

**Affiliations:** 1 UOC Medical Genetics, AORN A. Cardarelli, 80131 Naples, Italy; marina.tarsitano@aocardarelli.it; 2 Department of Medical Genetics, Clinical Analysis Center, Medicina Futura, Via Alcide De Gasperi, 107, Acerra, 80011 Naples, Italy; 3 UOC of Neurology and Stroke Unit, AORN A. Cardarelli, 80131 Naples, Italy; vincenzo.andreone@aocardarelli.it (V.A.);; 4 Division of Internal Medicine 2, Department of Medicine and Medical Specialties, AORN A. Cardarelli, 80131 Naples, Italy; maria.bova@aocardarelli.it (M.B.);; 5 Department of Translational Medical Sciences, WAO Center of Excellence, University of Naples Federico II, 80131 Naples, Italystefania.loffredo2@unina.it (S.L.); 6 Center for Basic and Clinical Immunology Research (CISI), University of Naples Federico II, 80131 Naples, Italy; 7 Italian Network for Hereditary and Acquired Angioedema (ITACA), 80131 Naples, Italy; 8 Complex Operative Unit of Pathological Anatomy and Molecular Diagnostics, San Giovanni di Dio Ruggi d’Aragona, Salernitan Medical School, 84121 Salerno, Italy

**Keywords:** alteplase-associated angioedema, contextual pathogenicity, precision medicine, clinical-exome sequencing

## Abstract

Intravenous thrombolysis with recombinant tissue-type plasminogen activator (tPA) remains a keystone of acute ischemic stroke treatment but in a subset of patients is complicated by angioedema, a potentially life-threatening adverse event largely mediated by bradykinin signaling. The unpredictable and idiosyncratic nature of this reaction has long suggested an underlying genetic contribution, yet its molecular architecture has remained poorly characterized. We hypothesized that alteplase-associated angioedema represents a multigenic susceptibility phenotype, arising from the convergence of rare genetic variants across multiple interacting physiological systems rather than from a single causal variant. To explore this hypothesis, we performed clinical exome sequencing in a cohort of 11 patients who developed angioedema following alteplase administration. Rather than identifying a shared pathogenic variant, we observed distinct yet convergent patterns of genetic vulnerability, allowing patients to be grouped according to dominant, but overlapping, biological axes. These included alterations affecting bradykinin regulation (e.g., *ACE*, *SERPING1*, *XPNPEP2*), endothelial structure and hemostasis (e.g., *VWF*, *COL4A1*), neurovascular and calcium signaling (e.g., *SCN10A*, *RYR1*), and vascular repair or remodeling pathways (e.g., *PSEN2*, *BRCA2*). Notably, many of the identified variants were classified as Variant of Uncertain Significance (VUS) or likely benign significance in isolation. However, when considered within an integrated, pathway-based framework, these variants can be interpreted as capable of contributing cumulatively to system level fragility, a phenomenon best described as “contextual pathogenicity”. Under the acute biochemical and proteolytic stress imposed by thrombolysis, this reduced physiological reserve may allow otherwise compensated vulnerabilities to become clinically manifest. Together, these findings support a model in which severe alteplase-associated angioedema appears as an emergent property of interacting genetic networks, rather than a monogenic disorder. This systems level perspective underscores the limitations of gene centric interpretation for adverse drug reactions and highlights the potential value of pathway informed, multi-genic approaches to risk stratification. Such frameworks may ultimately contribute to safer, more personalized thrombolytic decision, while providing a conceptual foundation for future functional and translational studies.

## 1. Introduction

**Figure 1:**
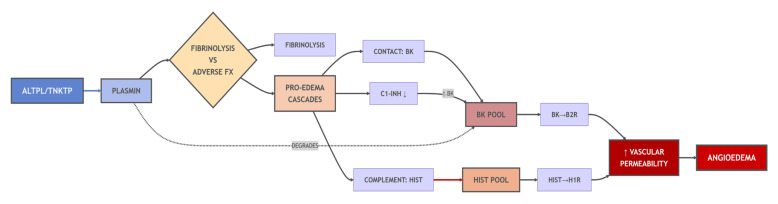
Pathway of thrombolytic-induced angioedema. Thrombolytic agents (Alteplase/Tenecteplase) activate plasmin, which mediates therapeutic fibrinolysis but also triggers adverse pro-edema cascades. These include activation of the contact system (releasing bradykinin/BK), the complement system (releasing histamine/HIST), and depletion of the C1-inhibitor (C1-INH ↓), which removes a key regulatory brake. Bradykinin and histamine synergistically activate vascular B2 and H1 receptors, increasing vascular permeability. Plasmin also degrades BK, creating a dynamic balance. The net effect of these amplified pathways is the clinical manifestation of angioedema. This diagram is conceptually aligned with the t-PA/plasminogen activation pathway described by Napolitano and Montuori [3]. Down arrow (↓) at C1-INH: Indicates that plasmin cleaves and inactivates C1-inhibitor, causing its levels to decrease. Up arrow (↑) at BK: Shows that reduced C1-INH activity results in excessive bradykinin (BK) generation and accumulation, which drives the pro-edema pathway through B2 receptor activation.

Acute ischemic stroke remains a major global contributor to both mortality and long-term disability. The cornerstone of its treatment is rapid reperfusion of the ischemic cerebral tissue in order to salvage neuronal tissue and preserve neurological function [1]. In this urgent setting, alteplase, a recombinant tissue-type plasminogen activator (t-PA, CAS: 105857-23-6; PubChem: 17396994), is a well-established pharmacologic treatment for intravenous thrombolysis. Engineered to replicate the structure and function of endogenous t-PA, alteplase facilitates the conversion of plasminogen to plasmin, initiating fibrin degradation and thereby promoting clot dissolution. Its high fibrin affinity directs plasminogen activation specifically at the thrombus interface, a characteristic that mitigates systemic fibrinolysis and reduces hemorrhagic risk compared with earlier, less selective agents [2]. (Figure 1).

Clinically, timely administration of alteplase can distinguish between restoration of function and permanent disability. Consequently, international societies as American College of Cardiology (ACC), American Heart Association (AHA) and European Society of Cardiology (ESC), prescribe stringent protocols for its use, bounding inclusive criteria within narrow therapeutic windows (for example, ≤4.5 h in ischemic stroke) and defining close surveillance for hemorrhagic complications, especially intracranial hemorrhage, the most feared adverse event [4]. Although newer fibrinolytic agents such as tenecteplase (CAS: 191588-94-0; PubChem: 47207728) offer considerable logistical advantages (e.g., longer half-life and single-bolus administration), they have not yet fully replaced alteplase in all clinical settings. This is due to a combination of factors, including variations in clinical evidence (e.g., optimal dosing, subgroups) and regulatory or institutional adoption challenges, although these barriers are increasingly being addressed [5]. Pharmacologic reperfusion with alteplase can restore perfusion and reduce thrombotic burden, contributing to improved clinical outcomes [6].

A rare but serious complication of thrombolysis with alteplase is angioedema, a condition characterized by sudden, localized swelling of deep dermal and mucosal tissues, commonly affecting the face, lips, tongue, palate or larynx. Unlike urticaria, which involves superficial dermis and is often pruritic, angioedema arises from subcutaneous or submucosal fluid extravasation, typically non-pruritic but potentially painful or tense [7]. In the setting of acute ischemic stroke treated with alteplase, the incidence of angioedema has been estimated at 1–5%. While uncommon, involvement of the oropharyngeal or laryngeal structures can precipitate airway obstruction, requires immediate recognition and intervention [8].

Mechanistically, alteplase induced angioedema is non-IgE-mediated and predominantly related to activation of the bradykinin pathway. Alteplase converts plasminogen into plasmin which can induce bradykinin generation through kallikrein-kinin system activation. Bradykinin exerts potent vasodilatory and permeability-increasing effects by prompting endothelial contraction and intercellular gap formation, thereby facilitating plasma extravasation into the interstitial compartment. In parallel, plasmin may activate the complement cascade, augmenting inflammatory mediator release and further enhancing vascular leak [9,10].

Targeted therapies for bradykinin-mediated angioedema, including plasma kallikrein inhibitors, such as ecallantide, C1-esterase inhibitor concentrates, and the bradykinin B2-receptor antagonist icatibant, have been used in several centers to manage alteplase-associated angioedema (AAAE), despite being off-label in this setting. Their use reflects the growing evidence, summarized in recent reviews, that AAAE often fails to respond to standard treatments such as antihistamines, corticosteroids, and epinephrine, which are effective for histaminergic angioedema but not for bradykinin-driven disease [11].

In line with this emerging evidence, the latest guidelines for the management of acute ischemic stroke acknowledge icatibant and C1-esterase inhibitor as possible alternatives for orolingual angioedema occurring during intravenous alteplase infusion. However, the guidelines do not specify when these agents should be administered, drawing largely on their efficacy in hereditary angioedema (HAE) and ACE-inhibitor induced angioedema rather than on data specific to AAAE [12].

This gap highlights the urgent need for additional studies and for a practical flowchart that neurologists and intensivists can use when facing this potentially life-threatening complication. As airway compromise may progress rapidly, endotracheal intubation or tracheostomy may become necessary, emphasizing the need for early recognition and escalation of care.

The wide interindividual variability observed in susceptibility to AAAE prompted us to consider whether genetic vulnerability may influence the risk of developing this adverse reaction. A natural conceptual link is the existence of HAE, including forms with normal C1-inhibitor activity, which demonstrates how monogenic or multi-genic defects in the bradykinin pathway can predispose otherwise healthy individuals to angioedema. Consistent with this framework, we systematically excluded the presence of pathogenic variants in all genes currently recognized as being associated with HAE by means of comprehensive sequencing analyses [13]. This finding supports the exclusion of classical monogenic forms of HAE and suggests alternative genetic or regulatory mechanisms underlying the observed phenotype.

Previous studies have demonstrated that genetic variant in multiple components involved in the regulation of bradykinin production and degradation, specifically *ACE*, *XPNPEP2*, *KLKB1*, *SERPING1*, *F12*, may modulate individual susceptibility to bradykinin-mediated angioedema. These genes encode enzymes, receptors, or regulatory proteins that play critical roles in peptide metabolism, bradykinin receptor signaling, and the control of the kallikrein-kinin cascade.

Functionally relevant variants within these loci can impair bradykinin degradation, enhance its generation, or alter receptor responsiveness, thereby disrupting the physiological balance of the kallikrein-kinin system. Such perturbations shift the system toward a state of bradykinin excess, ultimately increasing vascular permeability and predisposing affected individuals to the development of angioedema episodes [14].

The aim of the present study is therefore to clarify the genetic architecture underlying alteplase-associated angioedema (AAAE) through clinical exome sequencing (CES). By analyzing approximately 5500 clinically relevant genes in patients who experienced AAAE, this study aimed to achieve several interconnected objectives: to identify recurrent or novel genetic variants that predispose individuals to this condition; to assess whether a genetic risk-stratification model could differentiate between higher-risk and lower-risk patients; to investigate potential polygenic contributions to the initial ischemic event; and to define candidate genetic biomarkers for use prior to treatment. These biomarkers could advise precision medicine strategies aimed at minimizing the risk of severe adverse reactions to thrombolytic therapy. This research supports the main principles of predictive, personalized, and preventive medicine, with the ultimate goals of enhancing the safety of acute stroke management and advancing the comprehension of bradykinin-mediated angioedema. In addition, the primary contribution of this work is conceptual and hypothesis-generating: we use CES data to propose a system-level, multigenic susceptibility framework for AAAE, rather than a definitive variant-level suggestion model.

## 2. Methods

### 2.1. Patient Cohort and Phenotypic Characterization

Between December 2021 and July 2022 (determined by institutional ethics approval duration and funding period), a total of 198 patients with acute ischemic stroke received intravenous alteplase (R-tPA) at the Neurology and Stroke Unit of A.O.R.N. Cardarelli (Naples, Italy). Among them, 11 subjects (5.56%) developed AAAE, forming the case cohort of this study. Angioedema typically involved the tongue, lips, or eyelids, with onset occurring 30–180 min after the start of thrombolysis. In all cases, alteplase infusion was completed without interruption, consistent with current clinical practice. Episodes were self-limiting, with resolution occurring between 2 and 96 h; 5 of the 11 patients received corticosteroids and/or antihistamines as symptomatic treatment. The study was conducted in accordance with the Declaration of Helsinki, and all participants provided written informed consent. Clinical evaluation revealed typical stroke risk factors, including antihypertensive therapy with ACE inhibitors or ARBs (Angiotensin II Receptor Blocker, Novartis AG, Basel, Switzerland) in 7/11 patients, highlighting potential pharmacogenetic interactions. To exclude hereditary or acquired C1-inhibitor deficiency, all patients underwent complement testing. C1-esterase inhibitor antigenic levels, functional activity, and C4 concentrations were within the normal range, ruling out known forms of hereditary or acquired angioedema and supporting the classification of these cases as drug-induced angioedema [10].

### 2.2. Genomic DNA Extraction

Genomic DNA was isolated from peripheral blood samples collected in K_3_-EDTA vacuum tubes. Extraction was performed using the MagCore^®^ Genomic DNA Whole Blood Kit (RBC Bioscience Corp., Xindian District, New Taipei City, Taiwan) automated nucleic acid extractor.

### 2.3. Clinical Exome Sequencing and Primary Data Processing

Library preparation and CES were performed using the SOPHiA GENETICS’ Clinical Exome Solution v3 (SOPHiA GENETICS SA, Rolle, Switzerland), targeting approximately 5500 clinically genes. Sequencing was carried out on an Illumina NextSeq 550 platform following the manufacturer’s standard protocols, ensuring a minimum of 30× coverage for >95% of the target regions. (https://www.sophiagenetics.com/sophia-ddm-for-genomics/rare-disorders/, accessed on 29 June 2025). The complete list of genes captured by the Clinical Exome Solution v3 panel (https://www.sophiagenetics.com/wp-content/uploads/2024/09/CESv3-Factsheet.pdf, accessed on 29 June 2025) is provided as Supplementary S1. All sequencing procedures, bioinformatic pipelines, and variant interpretation workflows were rigorously conducted in compliance with current American College of Medical Genetics and Genomics/Association for Molecular Pathology (ACMG/AMP) guidelines and the Genome Analysis Toolkit (GATK) Best Practices for germline NGS analysis. Sequencing and primary data processing followed a clinically validated workflow that is routinely implemented in our diagnostic laboratory for daily reporting, ensuring high analytical sensitivity and specificity.

### 2.4. Bioinformatic Processing on the SOPHiA DDM Platform

FASTQ files were analyzed using the SOPHiA DDM (Data-Driven Medicine) platform (https://www.sophiagenetics.com/sophia-ddm/, accessed on 29 June 2025), which employs a standardized and validated bioinformatic pipeline. First, sequencing reads were aligned to the human reference genome (GRCh38/hg38) using the BWA-MEM algorithm, which is well-regarded for its accuracy even with variable-length sequences. Next, PCR duplicates were identified and marked with Picard Tools to prevent potential artifacts from affecting variant detection. Finally, genetic variants, including Single Nucleotide Variants (SNVs) and small insertions or deletions (Indels), were called using GATK HaplotypeCaller, ensuring high-fidelity detection of genetic variation. At the time of analysis, the core open-source components integrated within the SOPHiA DDM pipeline corresponded to BWA-MEM v0.7.17, Picard v2.27.5, and GATK HaplotypeCaller v4.2.6.1, all maintained and deployed by SOPHiA GENETICS within a continuously updated and quality-controlled environment. Details about patient variants are included in Supplementary S2. Raw sequencing data (FASTQ files), intermediate alignment files (BAM), and final variant call format (VCF) files are securely stored on the SOPHiA DDM platform and mirrored on institutional servers.

### 2.5. Variant Annotation, Filtration, and Analysis Strategy

Identified variants were annotated by integrating data from multiple international genomic databases (e.g., ClinVar, dbSNP, OMIM, gnomAD) to provide functional, frequency, and clinical context. Our analysis employed a dual-strategy approach on the SOPHiA DDM platform:

A targeted in silico search of the Sophia platform using the discrete term “angioedema” retrieved a curated gene panel enriched for variants with validated human phenotypic associations across hereditary and non-hereditary angioedema subtypes. The panel comprises 29 genes: *ACE*, *ANG*, *ANGPTL3*, *ANGPTL4*, *ANGPTL5*, *ANGPTL6*, *C1QA*, *C1QB*, *CLEC3B*, *FLT1*, *FLT3*, *FLT4*, *F12*, *KDR*, *KLK1*, *KLKB1*, *KNG1*, *MYO1F*, *MYO5B*, *PLAU*, *PLG*, *SERPING1*, *TEK*, *VEGFA*, *VEGFC*, *ETV6*, *MME*, *KCNMA1*, *CPN1*, and *XPNPEP2*.

Multi-genic Analysis: To move beyond known associations and enable novel gene discovery, we implemented a custom, hypothesis-driven multi-genic analysis. This approach applied systematic bioinformatic filters to distill thousands of initial variants down to a high-priority candidate gene list, based on two pillars of rare disease genetics:

• Pathogenicity: Retention of variants with a predicted damaging impact (e.g., missense, frameshift, splice-site).
• Rarity: Application of a Minor Allele Frequency (MAF) filter of <0.05% in the gnomAD database, a threshold chosen to capture variants with potential incomplete penetrance.

Furthermore, we excluded variants following a strict autosomal recessive (AR) inheritance model. The AAAE phenotype, manifesting in heterozygous individuals without reported homozygosity, is biologically incompatible with a pure AR mechanism, which requires biallelic loss-of-function and presents a distinct familial pattern not observed in our cohort [15]. In addition to population-based filters, all candidate variants were cross-checked against our internal database of >900 CES generated with the same platform and analytic workflow. This internal reference resource enabled comparative verification that prioritized variants exhibit extremely low frequencies not only in gnomAD but also in our local population, thereby reducing the likelihood that they represent benign background polymorphisms. Variants with elevated frequency in the internal database were excluded from further consideration.

### 2.6. Gene Prioritization

The refined gene lists were exported for external bioinformatic validation. A systematic text-mining strategy was employed using the DisGeNET platform (https://disgenet.com/), querying with a precise semantic grid of keywords (“angioedema”, “ischemic stroke”, “vascular permeability”, “bradykinin”, “drug resistance”) to identify genes with documented associations relevant to our phenotype and the pharmacological action of Alteplase [16].

Subsequently, a multi-level functional characterization pipeline was established to comprehensively evaluate the prioritized candidate variants. This integrative approach combined multiple specialized databases and in silico prediction tools, including:

Franklin by Genoox (https://franklin.genoox.com/clinical-db/home, accessed on 29 June 2025) and VarSome (https://varsome.com/), both used to obtain up-to-date clinical annotations and automated classifications according to American College of Medical Genetics and Genomics (ACMG) and the Association for Molecular Pathology (AMP) guidelines [17,18].

Genome Aggregation Database (gnomAD; https://gnomad.broadinstitute.org), employed to assess population allele frequencies and to derive CADD (Combined Annotation Dependent Depletion) scores [19,20]. AlphaMissense (https://alphamissense.hegelab.org/), used for in silico prediction of variant deleteriousness through advanced deep learning–based models [21].

In addition, ClinVar (https://www.ncbi.nlm.nih.gov/clinvar/, accessed on 29 June 2025) was queried to cross-reference the identified variants with previously reported clinical interpretations [22].

**Figure 2:**
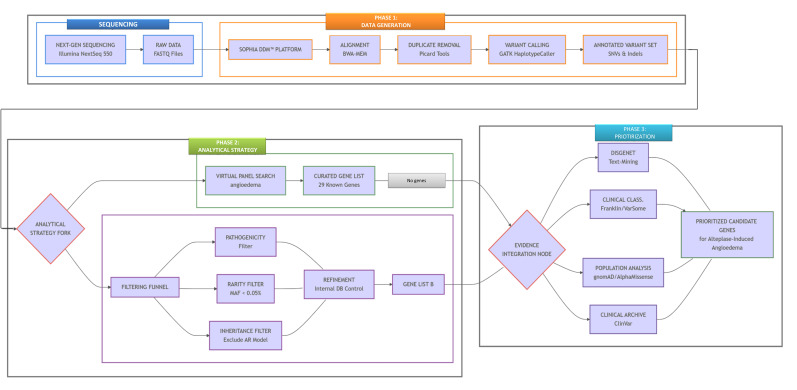
This figure illustrates the three-phase genomic analysis pipeline for identifying Alteplase-induced angioedema susceptibility, proceeding from left to right. Phase 1 initiates with high-coverage Clinical Exome Sequencing on an Illumina platform, which generates raw FASTQ data that undergoes processing through the validated SOPHiA DDM™ pipeline to perform genome alignment, artifact removal, and variant calling, ultimately producing an annotated variant set. Phase 2 implements a dual analytical strategy through two parallel approaches: a targeted query of 29 known angioedema-associated genes, and a discovery-mode systematic filter applying stringent pathogenicity, rarity (MAF < 0.05%), and autosomal recessive exclusion criteria to identify novel candidates. Phase 3 converges both gene sets for validation through multi-source evidence evaluation across specialized databases (DisGeNET for disease associations, Franklin/VarSome for clinical classification, gnomAD/AlphaMissense for population frequency and predictive scoring, and ClinVar for existing clinical reports) synthesizing these evidence layers into a final list of high-confidence prioritized candidate genes.

This comprehensive and integrative strategy enabled a robust evaluation of the clinical and functional relevance of the detected variants, emphasizing those with the highest likelihood of contributing to susceptibility to Alteplase-induced angioedema and/or the index ischemic stroke (Figure 2).

## 3. Results

### 3.1. Integrative Multi-Genic Analysis of Acute Angioedema Following Thrombolytic Therapy

**Figure 3:**
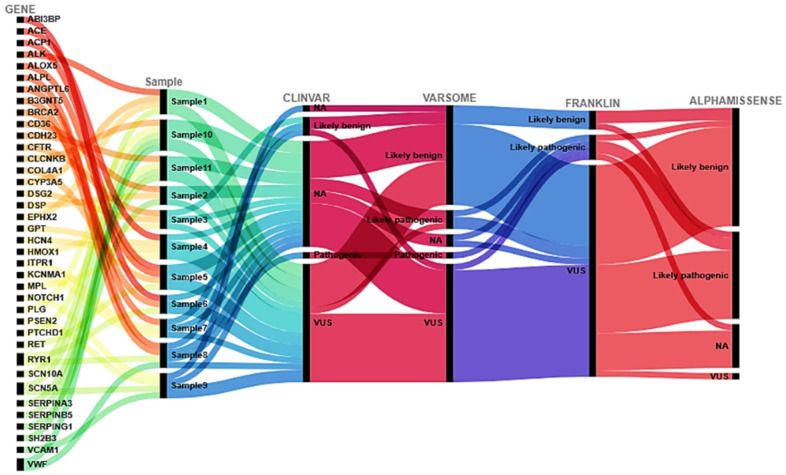
Alluvial plot illustrating the flow of gene variants across multiple annotation and pathogenicity-classification platforms. The leftmost column lists the genes in which variants were identified. Each gene connects to one or more patient samples (central “Sample” column), indicating the presence of specific variants within those samples. Subsequent columns represent the categorical classifications assigned by four independent interpretation tools (VarSome, ClinVar, Franklin, and AlphaMissense). Within each platform, variants are classified into standard clinical significance categories (e.g., likely benign, VUS, likely pathogenic, pathogenic, or not available [NA]). The width of each ribbon is proportional to the number of variants shared between categories, enabling direct visualization of concordance and discrepancies among classification systems. Overall, the diagram highlights the variability of variant interpretation across tools, emphasizing areas of agreement as well as reclassification trajectories suggestive of interpretive uncertainty or potential clinical relevance.

In this exploratory study, we performed an integrative multi-genic analysis in a cohort of eleven patients who developed AAAE. Although our findings are preliminary and cannot establish causality, the overall pattern supports the view that the phenotype may not stem from single gene Mendelian defects. Instead, it is more consistent with a form of multigenic vulnerability, potentially arising from the hypothetical convergence of several biologic domains including bradykinin-related enzymatic cascades, endothelial and hemostatic regulation, and craniofacial neurogenic innervation and signaling. Within this conceptual framework, low-penetrance variants and Variant of Uncertain Significance (VUS) could hypothetically act as modulators that, when co-occurring in functionally related pathways, might lower the physiological threshold for vascular barrier instability during the combined stress of acute cerebral ischemia and thrombolytic-induced proteolytic activation (Figure 3).

### 3.2. Stratification of Patients into Discrete Pathophysiological Clusters

The genomic pattern observed across these patients is more consistent with a distributed architecture of susceptibility than with isolated, deterministic genetic lesions, revealing its collective biological relevance only when exposed to the extreme biochemical and mechanical stress imposed by ischemia and thrombolytic therapy.

In the first patient, we identified a rare missense variant in *SCN10A* (c.4291G>A), classified as a variant of uncertain significance (VUS), which may contribute to a state of neurogenic hyper-reactivity [23]. This variant co-occurs with an intronic alteration in *EPHX2* (c.1083+4A>G), also classified as a VUS, for which no direct experimental evidence currently indicates an effect on splicing or enzymatic activity. *EPHX2* encodes soluble epoxide hydrolase, a key enzyme involved in the regulation of epoxyeicosatrienoic acid metabolism [24].

When interpreted in an integrated manner, together with additional rare variants affecting pathways related to xenobiotic metabolism and intracellular signaling, specifically involving *CYP3A5* and *ALK*, these genetic findings collectively remain classified as VUS. Notably, all identified variants displayed CADD scores exceeding 20, consistent with a high likelihood of functional relevance, and were observed at extremely low frequencies in the general population, with minor allele frequencies below 0.01% in the gnomAD database [25,26]. In our internal cohort, these variants were either absent or present at exceptionally low frequencies, further underscoring their rarity within the local population.

In the second patient, susceptibility appears to follow a more coherent biochemical trajectory, anchored by a pathogenic frameshift variant in *CD36* (c.1155dupA), a gene with well-established roles in lipid transport, oxidative stress sensing, and endothelial signaling [27]. This alteration co-occurs with rare missense variants in *SERPING1* (c.857G>A) and *DSP* (c.6575G>A), both classified as VUS. Although neither variant has been conclusively demonstrated to directly disrupt protein function, we hypothesize that their convergence within pathways governing protease inhibition and intercellular structural. Consistent with this interpretation, both the *SERPING1* and *DSP* variants exhibit CADD scores exceeding 20, indicative of a predicted deleterious impact in the absence of definitive functional validation. In contrast, the *CD36* frameshift variant was classified as pathogenic, reflecting its predicted loss-of-function consequence and established biological relevance [28,29].

The genetic profile of the third patient suggests a potential disequilibrium among oxidative stress regulation, epithelial–endothelial cohesion, and ion transport, shaped by rare variants in *HMOX1* (c.836C>T), *CFTR* (c.1795A>G), and *DSG2* (c.1781T>C). Each of these variants is individually classified as a VUS, and none is sufficient, in isolation, to support a definitive inference of functional disruption. Nevertheless, it is hypothesized that all three variants, which exhibit CADD scores ranging from 20.3 to 22.1, might be consistent with a predicted deleterious potential in the absence of direct functional validation [30,31,32].

The fourth patient illustrates an assemblage of rare genetic variants that collectively converge on pathways regulating bradykinin signaling, vascular tone, and endothelial responsiveness, rather than pointing to a single, clearly causative lesion. The most notable finding is a missense variant in *ACE* (c.1420T>C; p.Trp474Arg), classified as a VUS and characterized by a high CADD score. Although functional data are not currently available to demonstrate a direct effect of this substitution on angiotensin-converting enzyme activity, *ACE* is well established as a central regulator of bradykinin degradation and vascular homeostasis [33]. This potential susceptibility may be further shaped by the presence of the *HCN4* variant c.3599C>T (p.Pro1200Leu). While classified as likely benign in population databases, *HCN4* encodes a hyperpolarization-activated cyclic nucleotide–gated channel that plays a recognized role in cardiac pacemaker activity and has been implicated in the regulation of vascular smooth-muscle excitability [34]. Additional contributions may arise from the rare *MPL* variant c.1249G>A (p.Ala417Thr), affecting the thrombopoietin receptor, which is involved in hematopoietic signaling and vascular biology [35]. Finally, the *GPT* variant c.194G>A (p.Arg65His) impacts alanine aminotransferase, a metabolic enzyme increasingly regarded not only as a biomarker but also as a potential mediator of endothelial metabolic stress [36].

In the fifth patient, the genetic profile points toward a structural inflammatory susceptibility pattern, with variants converging on extracellular matrix integrity, growth-factor signaling, and inflammatory modulation. Central to this pattern is the *COL4A1* c.1454C>T (p.Pro485Leu) variant. *COL4A1* encodes a core component of the vascular basement membrane [37]. An increased inflammatory susceptibility may be further implied by the presence of the rare *ALOX5* missense variant c.178G>A (p.Glu60Lys), which affects a gene that plays a central role in leukotriene biosynthesis and inflammatory signaling [38]. Additional modulation may derive from *ACP1* c.97G>A (p.Asp33Asn), a variant impacting a low–molecular-weight protein tyrosine phosphatase involved in insulin and growth factor–mediated signaling pathways [39]. Finally, the *RET* variant c.785T>C (p.Val262Ala), characterized by a high CADD score, affects a receptor tyrosine kinase with established roles in cell survival, differentiation, and tissue remodeling, further supporting the biological plausibility of a pro-inflammatory and remodeling-prone molecular context [40].

The molecular profile identified in the sixth patient suggests a potential enrichment of biological pathways related to calcium-dependent signaling, maintenance of genomic integrity, and extracellular matrix–mediated cell–cell communication. In this context, we hypothesize that the observed pattern, encompassing rare genetic variants in *RYR1* (c.10648C>T), *BRCA2* (c.7972T>C), and *ABI3BP* (c.1608G>C), may collectively contribute to disease susceptibility [41,42,43].

The seventh patient displays a genetic profile in which alterations affecting electrophysiological control and calcium-dependent signaling pathways appear to be the prevailing features. The most prominent finding is the *KCNMA1* missense variant c.1613C>T (p.Pro538Leu), classified as a VUS but associated with a high CADD score, suggesting a potential functional impact [44]. This putative effect may be further reinforced by the presence of the rare *ITPR1* variant c.4543G>A (p.Gly1515Ser), which affects the inositol 1,4,5-trisphosphate receptor type 1, a critical mediator of intracellular calcium release from the endoplasmic reticulum [45]. An additional level of biological complexity is introduced by the *ANGPTL6* truncating variant c.887G>A (p.Trp296*), also classified as a VUS. *ANGPTL6* plays an important role in angiogenesis, endothelial metabolism, and the maintenance of vascular homeostasis [46].

The eighth patient exhibits a more complex convergence of calcium signaling, peptide regulation, and endothelial structural modulation, centered on a rare *RYR1* variant and shaped by additional potential genetic modifiers. The *RYR1* missense variant c.7921C>T (p.Leu2641Phe), classified as a VUS but predicted as likely pathogenic by several in silico algorithms, affects the ryanodine receptor, a principal intracellular calcium-release channel with broad physiological relevance [47]. A key additional finding in this patient is the pathogenic *ALPL* variant c.571G>A (p.Glu191Lys). *ALPL* encodes tissue-nonspecific alkaline phosphatase, an enzyme that plays a central role in maintaining purinergic signaling homeostasis and supporting normal endothelial function [48]. Further modulation may be contributed by *B3GNT5* c.871A>G (p.Asn291Asp), a rare missense variant affecting glycosphingolipid biosynthesis. Although currently classified as likely benign, glycosphingolipids are integral components of membrane microdomains and are involved in receptor clustering, including receptors implicated in inflammatory responses and vascular permeability regulation [49]. Finally, it is plausible that the *VWF* variant c.3692A>C (p.Asn1231Thr), despite its classification and relatively higher allele frequency, may add a structural and hemostatic dimension to the patient’s molecular profile, thereby contributing to the overall vascular phenotype [50].

The molecular profile of the ninth patient suggests a background of chronic endothelial vulnerability, characterized by the presence of rare variants in *PSEN2* (c.766del), *NOTCH1* (c.1838G>A), *VCAM1* (c.353A>T), and *SCN5A* (c.4853C>T). While these variants are predominantly classified as VUS or likely benign, they occur at low population frequencies and affect genes with well-established roles in endothelial integrity, cell–cell signaling, vascular adhesion, and electrophysiological regulation, respectively [51,52,53,54].

In the tenth patient, a pattern centered on coagulation and intracellular signaling becomes apparent. This profile includes rare variants in *PLG* (c.1748G>A), *VWF* (c.974G>T), and *SH2B3* (c.364G>A), together with intronic alterations affecting *CLCNKB* and *SCN5A*. The convergence of these variants on pathways regulating fibrinolysis, hemostasis, and immune-vascular signaling suggests a coordinated perturbation of coagulative and signaling homeostasis [55,56,57,58].

Finally, the eleventh patient exemplifies the concept of multi-genic sensitization, harboring rare variants in *PTCHD1* (c.2396T>C), *CDH23* (c.457G>A), *SERPINB5* (c.547T>C), and *SERPINA3* (c.338C>T). All identified variants were classified as VUS or likely benign; however, their CADD scores, ranging from 19.8 to 22.3, are consistent with modest to potentially deleterious predicted functional effects. In the absence of definitive evidence for pathogenicity, this aggregate genetic burden nonetheless supports a model in which multiple low-impact variants may collectively contribute to disease susceptibility [59,60,61,62].

### 3.3. Integrative Analysis of Shared Genomic Architecture Across Patients

**Figure 4:**
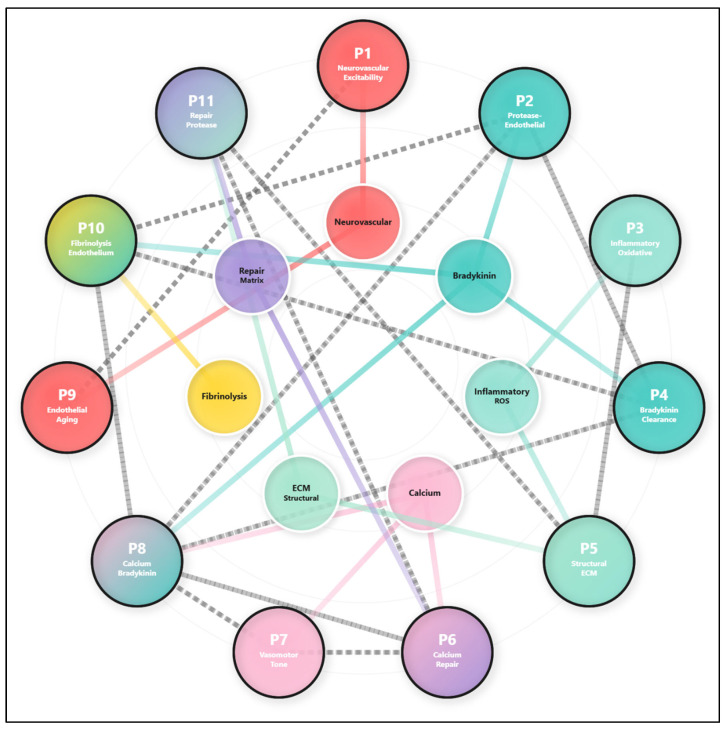
Network representation of patient-specific pathophysiological patterns and shared mechanistic axes. The diagram integrates eleven individualized vulnerability profiles (P1–P11), each anchored in distinct primary driver variants and modulated by secondary genetic contributors. Peripheral patient nodes map onto central mechanistic hubs neurogenic, bradykinin-mediated, inflammatory, endothelial, fibrinolytic, calcium-signaling, remodeling, and hedgehog-related pathways, reflecting convergent biological axes across the cohort. P1 displays a unique neurogenic hyper-reactivity signature driven by *SCN10A*, with modulators (*EPHX2*, *CYP3A5*, *ALK*) supporting a distinctive neuronal-endothelial coupling axis. P2, P4, and P8 cluster along a shared bradykinin-endothelial dysregulation axis, shaped by variants in *SERPING1*, *ACE*, *XPNPEP2*, and *VWF*, with further modulation by desmosomal, DNA-repair, metabolic, and ion-channel genes. P10 intersects this axis through combined PLG and VWF defects, linking fibrinolytic and endothelial instability. P3 defines a polygenic inflammatory/oxidative-stress signature driven by *HMOX1*, *CFTR*, and *IFNG*, sharing a broader inflammation-remodeling continuum with P5, whose remodeling failure arises from *COL4A1*, *ALOX5*, and *RET*. Calcium-signaling instability characterizes P6 (driven by *RYR1*) and overlaps with P8 through shared *RYR1* perturbation; both connect with P7 through a vasomotor dysregulation axis linked to *KCNMA1*. Neurovascular fragility is jointly represented by P9 and P11, involving *PSEN2*, *NOTCH1*, and hedgehog-pathway disruption via *PTCHD1*, with additional endothelial modulators (*VCAM1*, *CDH23*, *SERPINB5*, *SERPINA3*) reinforcing this shared axis. Together, the network highlights both distinct and intersecting genetic mechanisms shaping vascular, neurogenic, inflammatory, and endothelial vulnerability across patients.

When the genomic profiles described in Section 3.2 are considered collectively, a coherent integrative framework emerges in which alteplase-associated angioedema is best understood as the context-dependent expression of shared pathway-level vulnerabilities, rather than as the consequence of discrete or deterministic genetic lesions. The patients do not converge on identical variants, nor on single genes of large effect. Instead, they exhibit distinct combinations of rare genomic perturbations, most characterized by elevated CADD scores and variable inter-database classification, that repeatedly map to a restricted set of conserved biological axes governing vascular stability, neurovascular signaling, inflammatory balance, and bradykinin regulation (Figure 4).

Across the cohort, variants such as *SCN10A* (Patient 1), the pathogenic *CD36* frameshift (Patient 2), *ACE* (Patient 4), and *PLG* (Patient 10) illustrate this principle. None of these alterations implies monogenic causation, nor do they act in isolation. Rather, they occupy nodal positions within overlapping molecular circuits whose functional buffering capacity may become insufficient under the biochemical and proteolytic stress imposed by tissue plasminogen activator. The recurrence of specific pathway categories (e.g., intracellular calcium handling, endothelial cohesion, bradykinin clearance, inflammatory priming, and vascular repair) reflects a shared architectural logic of susceptibility rather than variant-level determinism.

The calcium-signaling axis provides a clear example of this integrative pattern. Rare *RYR1* variants (c.10648C>T in Patient 6 and c.7921C>T in Patient 8) affect a receptor central to intracellular Ca^2+^ release, yet their precise functional consequences remain unvalidated. When these variants are considered alongside additional rare alterations in *ITPR1* c.4543G>A and *KCNMA1* c.1613C>T (Patient 7), a convergent susceptibility pattern becomes apparent. Rather than directly lowering activation thresholds, these variants may collectively bias calcium-dependent endothelial excitability, increasing the likelihood that bradykinin-induced signaling and calcium flux become disproportionately coupled during thrombolysis, thereby favoring permeability over adaptive vasomotor responses.

A second recurrent axis involves endothelial structural integrity and barrier resilience. Variants in *VWF* (c.3692A>C in Patient 8 and c.974G>T in Patient 10), while classified as likely benign or VUS, affect a gene essential for shear-dependent endothelial interactions. In Patient 2, this background is accompanied by a pathogenic *CD36* frameshift, intersecting lipid handling and inflammatory modulation. Parallel rare variants in *COL4A1* (Patient 5) and *ABI3BP* (Patient 6) further illustrate distinct molecular routes toward modest reductions in structural and matrix-related resilience. These findings are most parsimoniously interpreted as cumulative modifiers of endothelial robustness, rather than as discrete causes of barrier failure.

Interfacing with these structural features is the bradykinin-regulatory axis, which recurs across patients through mechanistically heterogeneous variants. *SERPING1* (Patient 2), *ACE* (Patient 4), *XPNPEP2* (Patient 8), and *PLG* (Patient 10) intersect distinct steps of bradykinin generation, inhibition, or degradation. None of these variants independently establishes uncontrolled signaling; however, their distribution across the cohort supports a model in which bradykinin regulation becomes progressively less buffered, particularly during plasmin activation induced by alteplase.

Inflammatory and oxidative-stress pathways represent a fourth shared dimension. Rare variants in *ALOX5* (Patient 5), *HMOX1* (Patient 3), *VCAM1* (Patients 6 and 9), and *IFNG* (Patient 3) map to immune and redox regulatory circuits. Individually insufficient to dictate phenotype, these variants may collectively lower the threshold at which endothelial injury becomes self-reinforcing, particularly as oxidative stress intersects with calcium dysregulation and leukocyte adhesion during reperfusion.

A fifth and final axis centers on vascular remodeling and repair capacity. Rare variants in *PTCHD1* (Patient 11), *NOTCH1* (Patient 9), and *BRCA2* (Patient 6) span pathways governing endothelial differentiation, genomic maintenance, and recovery from oxidative damage.

Taken together, these axes define a cohesive but non-deterministic map of shared vulnerability, in which each patient expresses a personalized configuration of pathway level sensitivities.

## 4. Discussion

The rapid progress of NGS technologies has not simply enhanced the resolution of genetic diagnostics in clinical medicine; it has precipitated a fundamental epistemological shift in how genetic causality itself is conceptualized [63]. The classical Mendelian paradigm, centered on the identification of single, highly penetrant pathogenic variants, has proven increasingly inadequate for explaining complex and context-dependent phenotypes, particularly severe idiosyncratic adverse drug reactions [2]. Clinical exome analyses now consistently reveal a far more intricate landscape, one in which multiple rare variants, each exerting a modest and often ambiguous functional effect, converge across biological systems to undermine physiological resilience [64]. Disease susceptibility in this framework emerges not as a binary consequence of mutation presence or absence, but as a threshold phenomenon shaped by cumulative genomic burden interacting dynamically with environmental and pharmacological stressors.

This conceptual transition is exemplified by the present investigation into AAAE. The clinical and genetic features observed across the cohort resist reduction to categorical labels such as “pathogenic” or “likely benign,” nor can they be reconciled with a single causal genetic lesion. Instead, AAAE appears to arise from a distributed genomic architecture in which rare variants across multiple, interconnected biological pathways interact in a nonlinear and context-dependent manner to progressively erode vascular stability. In this setting, susceptibility is not encoded in any single gene, but rather emerges from the collective configuration of the genome under conditions of extreme physiological challenge.

Consistent with this model, variants identified in genes such as *SERPING1*, *ACE*, *XPNPEP2*, *RYR1*, *VWF*, *COL4A1*, *SCN10A*, *PTCHD1*, and others, many of which are classified as VUS, do not operate independently. Their biological relevance becomes apparent only when considered in aggregate and in relation to complementary physiological axes. Importantly, substantial discordance was observed among widely used variant interpretation platforms, including VarSome, ClinVar, Franklin, and AlphaMissense [19]. Far from undermining interpretability, this inconsistency is itself a hallmark of variants that participate in multifactorial phenotypes for which monogenic classification frameworks are intrinsically ill-suited. The absence of consensus classification reflects limited case-level evidence rather than biological irrelevance, and underscores the inadequacy of single-variant determinism when applied to emergent, system-level disorders [65].

Within this context, the use of CADD scoring served not as a surrogate for clinical pathogenicity, but as a prioritization tool grounded in probabilistic functional relevance [66]. A threshold score of 20, corresponding to the top 1% of predicted deleterious variants in the human genome, was employed to enrich for variants likely to perturb protein structure, splicing fidelity, or regulatory control [67]. Crucially, this approach acknowledges that functional impact does not equate to disease causation in isolation. Rather, variants exceeding this threshold represent plausible contributors within a multigenic architecture, particularly when distributed across interacting pathways and exposed to pharmacological stress such as thrombolysis.

Individual patient profiles illustrate how distinct constellations of variants bias vulnerability along specific physiological dimensions. Some patients exhibit a neurovascular excitability predisposition driven by ion channel and signaling variants that alter membrane excitability and calcium dynamics, thereby lowering the threshold for exaggerated vascular responses. Others display protease endothelial imbalance, in which inefficiencies in protease inhibition, lipid handling, or cytoskeletal integrity compromise the capacity of the endothelium to buffer thrombolytic stress. Additional profiles reveal inflammatory-oxidative susceptibility, impaired extracellular matrix repair, dysregulated vasomotor tone, or convergence of calcium signaling and bradykinin metabolism. In each case, no single variant is sufficient to account for the observed phenotype; rather, vulnerability arises from the coordinated weakening of multiple regulatory safeguards that normally preserve endothelial homeostasis (Table 1).

When these prioritized variants are analyzed across the entire cohort, a striking pattern emerges: genetic risk is not randomly distributed, but converges on three biologically coherent and tightly interconnected axes of vulnerability. These include the bradykininergic axis, governing permeability and inflammatory signaling, the endothelial-hemostatic axis, responsible for structural integrity, repair, and adaptive remodeling, the neurovascular signaling axis which integrates neural excitability and calcium mediated control of vascular tone. These systems do not function independently. Instead, they form an interdependent network in which perturbation of one axis can propagate dysfunction across the others, amplifying susceptibility under conditions of pharmacological challenge. The bradykininergic axis exemplifies this convergence with particular clarity. Variants affecting distinct biochemical steps, bradykinin degradation via *ACE*, alternative clearance through *XPNPEP2*, upstream modulation of plasmin generation by *PLG*, and regulatory control of kallikrein activity by *SERPING1*, do not individually induce uncontrolled signaling. However, their cumulative presence progressively diminishes buffering capacity. During alteplase-induced plasmin activation, this reduced regulatory reserve may permit bradykinin-mediated permeability responses to exceed physiological thresholds, precipitating angioedema in susceptible individuals. Parallel vulnerabilities are evident within the endothelial-hemostatic axis. Variants in genes such as *VWF*, *COL4A1*, *DSP*, *CD36*, *BRCA2*, and *ABI3BP* do not merely weaken the vessel wall mechanically. Rather, they intersect processes essential for active endothelial adaptation, including cytoskeletal remodeling, integrin-mediated adhesion, oxidative damage repair, and extracellular matrix turnover. In this framework, the endothelium is understood not as a passive structure subjected to injury, but as a dynamically stressed interface that must continuously integrate mechanical forces, inflammatory cues, and neurogenic signals during thrombolytic therapy. Equally critical is the contribution of the neurovascular signaling axis. Variants in sodium, potassium, and calcium channels, as well as associated signaling mediators, highlight how altered neural excitability and intracellular calcium handling can entrain endothelial behavior. Perturbations in calcium release or membrane excitability may lower the threshold for disproportionate vasomotor or permeability responses, even in the absence of overt structural fragility or primary defects in bradykinin metabolism. This insight expands the conceptual boundaries of angioedema susceptibility, situating it within a broader systems-level misalignment between neural signaling and vascular homeostasis. Across the cohort, certain genes repeatedly occupy nodal positions within these interacting networks, including *RYR1* in calcium instability, *VWF* in endothelial integrity, *PSEN2* in neurovascular remodeling, and *VCAM1* in inflammatory amplification. These nodes represent points of convergence where otherwise distinct pathogenic trajectories intersect. The most pronounced susceptibility profiles arise not from isolated defects, but from specific combinations of vulnerabilities, such as bradykinin dysregulation superimposed on compromised endothelial repair, or calcium signaling instability coupled with reduced structural resilience. This systems-level perspective necessitates a fundamental re-evaluation of the VUS category. Variants that appear clinically indeterminate in isolation may acquire functional and pathogenic relevance when embedded within a permissive genomic background and exposed to extreme pharmacological stress. In this sense, pathogenicity is not an intrinsic property of a variant, but an emergent feature of its biological context, a principle with profound implications for variant interpretation and clinical risk assessment. From a translational standpoint, these findings suggest that future strategies for risk stratification in thrombolytic therapy may derive greater value from integrated, pathway-based assessments of cumulative genetic burden than from conventional single-variant screening. The identification of convergent molecular hubs further highlights biologically plausible targets for therapeutic modulation, including attenuation of bradykinin signaling, stabilization of endothelial calcium homeostasis, and reinforcement of extracellular matrix integrity. Such approaches may ultimately enable more individualized preventive and therapeutic interventions for patients at elevated risk of AAAE. In this regard, our analytic and visualization strategy is conceptually aligned with contemporary multimodal genomic studies in oncology and hematology which integrate variant-level information, pathway context, and graphical representations to delineate complex genetic [68].

Several limitations must be acknowledged and framed within the appropriate epistemological context of this study. The absence of a prospectively sequenced, independent control group inherently precludes formal variant-level association testing and substantially limits causal inference. To mitigate this limitation, we compared all prioritized variants against our internal reference database, generated and analyzed with the same CES pipeline, thereby using this dataset as a comparator to confirm the rarity of candidate variants in our local population. Similarly, the relatively small cohort size restricts statistical power and precludes robust quantitative modeling of higher order gene to gene interactions, which are likely to contribute to complex, multifactorial vascular phenotypes. These constraints are intrinsic to the study design and necessitate a cautious interpretation of the genetic findings. Crucially, however, the purpose of the present investigation is not to establish deterministic or monogenic genetic causality. Rather, the primary objective of this work is to identify convergent biological vulnerabilities that may predispose individuals to adverse angioedematous events, particularly in the context of pharmacological exposure and acute cerebrovascular stress. The genetic variants identified in this study should therefore be conceptualized not as independently pathogenic entities, but as elements of a permissive or sensitizing biological landscape that may collectively lower the threshold for maladaptive or pathological responses under specific clinical or pharmacological conditions. Their contribution is best understood within a multifactorial framework in which genetic predisposition interacts with environmental, inflammatory, and therapeutic triggers rather than acting as a deterministic cause of disease. Elucidation of the mechanistic relevance of these variants will require dedicated functional investigation across complementary experimental systems. These include targeted cellular assays, iPSC-derived endothelial models that recapitulate vascular and inflammatory signaling dynamics [68], and in vivo platforms capable of integrating complex interactions among proteolytic cascades, inflammatory mediators, and vascular permeability pathways. Such integrative functional studies will be indispensable for rigorously testing the biological plausibility and synergistic nature of the mechanisms inferred from the genetic data, and for translating associative genomic signals into coherent pathophysiological insight. A further critical limitation is the absence of pre-thrombolysis bradykinin quantification, which prevents direct biochemical assessment of pharmacological priming effects associated with antecedent exposure to angiotensin-converting enzyme inhibitors or angiotensin receptor blockers. Importantly, this limitation does not reflect methodological insufficiency but arises from unavoidable constraints inherent to hyperacute stroke care. The clinical imperative of rapid reperfusion within a narrow therapeutic window precludes additional investigational blood sampling. Moreover, bradykinin’s extremely short half-life and marked ex vivo instability demand highly specialized collection and processing conditions that are incompatible with emergency clinical workflows [69]. Finally, acute cerebral ischemia itself triggers profound activation of endogenous inflammatory and kallikrein-kinin pathways [70], rendering any peri-treatment measurement biologically confounded by the ischemic insult. Consequently, although angiotensin receptor blockers may modulate bradykinin availability through alternative enzymatic or receptor-mediated mechanisms, the present study necessarily relies on indirect inference based on medication history rather than direct biochemical evidence of bradykinin priming. This limitation further reinforces the interpretative framework of the study, which is explicitly oriented toward vulnerability mapping rather than causal attribution. Only through the iterative integration of genomic data with functional experimentation and clinical phenotyping will it be possible to progress from associative signals to mechanistic insight, thereby enabling a more nuanced and biologically grounded approach to risk stratification and clinical decision-making in the era of precision medicine. Notwithstanding these limitations, the consistency of pathway-level convergence across eleven independently ascertained cases, combined with the robustness of a clinically validated NGS workflow and stringent rarity criteria applied both to public and internal control datasets, provides a solid foundation for the interpretive model proposed here.

## 5. Conclusions

This study proposes a refined conceptual framework for interpreting severe idiosyncratic drug reactions in the era of genome-scale analysis. Our findings support the view that susceptibility to AAAE is unlikely to be monogenic, and is more plausibly explained as a polygenic, systems-level phenomenon arising from the cumulative interaction of rare genetic variants across bradykinin signaling, endothelial integrity, neurovascular regulation, and inflammatory pathways. In this context, it is the configuration and interaction of variants, rather than the presence of any single alteration, that appears to influence whether vascular homeostasis is maintained or destabilized during thrombolytic stress.

By integrating variant-level observations, including *SERPING1*, *ACE*, *XPNPEP2*, *RYR1*, *VWF*, *COL4A1*, *PTCHD1*, among others, we illustrate how variants frequently classified as VUS or even likely benign significance may nonetheless contribute meaningfully to disease susceptibility when embedded within a permissive genomic background and exposed to a strong pharmacological trigger. This concept of contextual or emergent pathogenicity challenges strictly binary interpretative models and underscores the limitations of single-variant inference for complex adverse drug reactions. From a clinical perspective, these observations suggest that future risk stratification for thrombolytic therapy may benefit from integrated, pathway-oriented genetic assessments rather than isolated variant screening. Although immediate clinical implementation remains premature, the identification of convergent molecular axes and recurrent genetic hubs provides a foundation for developing predictive frameworks capable of flagging heightened vulnerability prior to alteplase exposure. In the longer term, such insights could inform more individualized therapeutic decision-making, including alternative reperfusion strategies or targeted prophylactic interventions aimed at mitigating bradykinin-mediated or endothelial instability.

More broadly, this work highlights a conceptual transition in precision medicine. Biological complexity should not be regarded as an impediment to interpretation, but rather as a source of explanatory power when approached with systems-level logic. Each patient represents a unique genomic configuration in which interacting variants shape physiological resilience under stress. Accordingly, the future of clinical genomics in adverse drug reaction research is likely to depend less on identifying solitary causal mutations and more on deciphering the networked architecture of genetic susceptibility, enabling a shift from reactive management toward more anticipatory, personalized, and mechanism-informed care.

## Figures and Tables

**Table 1:** Match Conservative Interpretation.

Patient	Dominant Pathophysiological Pattern	Primary Rare Variants (HGVS)	Additional Rare Modulators (HGVS)	Shared Axis/Overlap
**1**	Neurovascular excitability bias	*SCN10A* c.4291G>A	*EPHX2* c.1083+4A>G; *CYP3A5* c.188A>G; *ALK* c.1202G>A	Neuronal–endothelial signaling
**2**	Protease–endothelial imbalance	*CD36* c.1155dupA; *SERPING1* c.857G>A	*DSP* c.6575G>A; *XRCC1* c.1727A>C	Bradykinin/barrier axis
**3**	Inflammatory–oxidative susceptibility	*HMOX1* c.836C>T; *CFTR* c.1795A>G; *IFNG* c.–5A>G	*DSG2* c.1781T>C	Inflammation–ROS axis
**4**	Bradykinin clearance sensitivity	*ACE* c.1420T>C	*GPT* c.194G>A; *HCN4* c.3599C>T	Bradykinin axis
**5**	Structural ECM vulnerability	*COL4A1* c.1454C>T; *ALOX5* c.178G>A	*ACP1* c.97G>A	ECM–inflammation
**6**	Calcium and repair modulation	*RYR1* c.10648C>T	*BRCA2* c.7972T>C; *ABI3BP* c.1608G>C	Calcium/repair
**7**	Vasomotor tone modulation	*KCNMA1* c.1613C>T	*ITPR1* c.4543G>A; *ANGPTL6* c.887G>A	Calcium axis
**8**	Calcium–bradykinin convergence	*RYR1* c.7921C>T; *XPNPEP2* c.644C>T	*VWF* c.3692A>C; *ALPL* c.571G>A	Calcium/bradykinin
**9**	Endothelial aging profile	*PSEN2* c.766del; *NOTCH1* c.1838G>A	*SCN5A* c.4853C>T; *VCAM1* c.353A>T	Neurovascular integrity
**10**	Fibrinolysis–endothelium stress	*PLG* c.1748G>A; *VWF* c.974G>T	*SH2B3* c.364G>A; intronic CNVs	Bradykinin/fibrinolysis
**11**	Repair and protease regulation bias	*PTCHD1* c.2396T>C	*CDH23* c.457G>A; *SERPINB5* c.547T>C; *SERPINA3* c.338C>T	Repair/matrix balance

## Data Availability

All raw sequencing data (FASTQ files), alignment files (BAM), and variant call files (VCF), as well as detailed annotation outputs, are stored on the SOPHiA DDM platform and on secure institutional servers. Given the size and sensitive nature of these datasets, they are not deposited in an open public repository; however, they can be made available to investigators upon reasonable request to the corresponding author, subject to approval by the local ethics committee and in full compliance with applicable data protection regulations.

## Supplementary Materials

The following supporting information can be downloaded at: https://www.mdpi.com/article/10.3390/life16020200/s1.

## Abbreviations

*SCN10A*	Sodium Voltage-Gated Channel Alpha Subunit 10
*EPHX2*	Epoxide Hydrolase 2
*CYP3A5*	Cytochrome P450 Family 3 Subfamily A Member 5
*ALK*	Anaplastic Lymphoma Receptor Tyrosine Kinase
*CD36*	CD36 Molecule
*DSP*	Desmoplakin
*SERPING1*	Serpin Family G Member 1
*DSG2*	Desmoglein 2
*CFTR*	Cystic Fibrosis Transmembrane Conductance Regulator
*HMOX1*	Heme Oxygenase 1
*ACE*	Angiotensin I Converting Enzyme
*HCN4*	Hyperpolarization Activated Cyclic Nucleotide Gated Potassium Channel 4
*MPL*	MPL Proto-Oncogene, Thrombopoietin Receptor
*GPT*	Glutamic-Pyruvic Transaminase
*RET*	RET Proto-Oncogene
*ACP1*	Acid Phosphatase 1
*ALOX5*	Arachidonate 5-Lipoxygenase
*COL4A1*	Collagen Type IV Alpha 1 Chain
*RYR1*	Ryanodine Receptor 1
*BRCA2*	BRCA2 DNA Repair Associated
*ABI3BP*	ABI Family Member 3 Binding Protein
*KCNMA1*	Potassium Calcium-Activated Channel Subfamily M Alpha 1
*ANGPTL6*	Angiopoietin Like 6
*ITPR1*	Inositol 1,4,5-Trisphosphate Receptor Type 1
*ALPL*	Alkaline Phosphatase, Biomineralization Associated
*B3GNT5*	UDP-GlcNAc:BetaGal Beta-1,3-N-Acetylglucosaminyltransferase 5
*VWF*	Von Willebrand Factor
*PSEN2*	Presenilin 2
*SCN5A*	Sodium Voltage-Gated Channel Alpha Subunit 5
*NOTCH1*	Notch Receptor 1
*VCAM1*	Vascular Cell Adhesion Molecule 1
*PLG*	Plasminogen
*SH2B3*	SH2B Adaptor Protein 3
*CLCNKB*	Chloride Voltage-Gated Channel Kb
*PTCHD1*	Patched Domain Containing 1
*CDH23*	Cadherin Related 23
*SERPINB5*	Serpin Family B Member 5
*SERPINA3*	Serpin Family A Member 3
