# Supplementary material for: Alteplase and Angioedema: Can Clinical Exome Sequencing Redefine the Paradigm?
Source: Life (Basel). 2026 Jan 26;16(2):200. doi: 10.3390/life16020200 (PMC12942441; doi:10.3390/life16020200)
Supplement: Supplementary file 1 [file life-16-00200-s001.zip › Supplementary S1 genes.pdf]

GENE	Codon	Protein	Prediction. VARSOME	CLINVAR	PREDIZ. FRANKLIN	PREDIZ. ALPHAMISSENSE	Allele count (EXAC)		CADD
							EXOME	GENOME	
Sample 1									
SCN10A	c.4291G>A	p.(Asp1431Asn)	VUS	VUS	VUS	Patogenetica (0.746)	41/1461814	4/152140	27,3
EPHX2	c.1083+4A>G	p.?	Likely benign	NA	VUS	NA	1/1442190	0/149300	23,1
CYP3A5	c.188A>G	p.(Glu63Gly)	Likely benign	NA	VUS	Likely benign	1/1461494	0/152290	21,8
ALK	c.1202G>A	p.(Arg401Gln)	Likely benign	VUS	VUS	Likely benign	9/1461830	7/152294	21,7
Sample 2									
CD36	c.1155dupA	p.(Arg386Thrfs*3 2)	Likely Pathogenic	NA	Likely Pathogenic	Likely Pathogenic	8/1458474	2/151062	32
DSP	c.6575G>A	p.(Arg2192His)	Likely Pathogenic	VUS	VUS	Likely benign	27/1461892	0/152252	28,5
SERPING1	c.857G>A	p.(Arg286His)	Uncertain Significate	NA	VUS	Likely Benign	15/1461894	5/152206	23,4
Sample 3									
DSG2	c.1781T>C	p.(Leu594Pro)	Benign	Likely benign	Likely benign	Likely benign	514/1461874	57/152320	22,5
CFTR	c.1795A>G	p.(Thr599Ala)	VUS	NA	VUS	Ambiguous	NA	NA	NA
HMOX1	c.836C>T	p.(Ala279Val)	VUS	VUS	VUS	Likely benign	NA	NA	NA
Sample 4									
ACE	c.1420T>C	p.(Trp474Arg)	VUS	VUS	VUS	Likely pathogenic	5/1461864	1/152196	32
HCN4	c.3599C>T	p.(Pro1200Leu)	Benign	VUS	VUS	Likely pathogenic	19/1443358	8/152290	25,8
MPL	c.1249G>A	p.(Ala417Thr)	VUS	VUS	VUS	Likely benign	4/1461876	0/152234	23,8
GPT	c.194G>A	p.(Arg65His)	Likely benign	NA	Likely benign	Likely pathogenic	794/1460958	70/152310	23,3
Sample 5									
RET	c.785T>C	p.(Val262Ala)	benign	VUS	VUS	Likely pathogenic	308/1460294	39/152180	29,8
ACP1	c.97G>A	p.(Asp33Asn)	VUS	NA	VUS	Likely benign	41/1461404	7/150038	25,9
ALOX5	c.178G>A	p.(Glu60Lys)	Likely benign	NA	VUS	Likely benign	14/1461880	11/152194	24,6
COL4A1	c.1454C>T	p.(Pro485Leu)	Likely benign	VUS	VUS	Likely benign	43/1461404	20/152182	23,4
Sample 6									
RYR1	c.10648C>T	p.(Arg3550Trp)	VUS	Classif. contrastanti	Likely Pathogenic	Likely benign	202/1461680	18/152232	32
BRCA2	c.7972T>C	p.(Tyr2658His)	VUS	VUS	VUS	Ambiguous/ 1_pathogenic (0.776)	NA	NA	NA
ABI3BP	c.1608G>C	p.(Gln536His)	Likely benign	NA	VUS	NA	NA	NA	NA

Sample 7									
KCNMA1	c.1613C>T	p.(Pro538Leu)	VUS	VUS	VUS	Likely pathogenic	6/1461848	0/152332	29,3
ANGPTL6	c.887G>A	p.(Trp296*)	VUS	NA	VUS	NA	NA	NA	NA
ITPR1	c.4543G>A	p.(Gly1515Ser)	VUS	Likely benign	VUS	Likely benign	13/1461600	0/152318	22,3
Sample8									
RYR1	c.7921C>T	p.(Leu2641Phe)	VUS	VUS	VUS	Likely Pathogenic	4/1456852	2/152182	26,1
ALPL	c.571G>A	p.(Glu191Lys)	Pathogenic	Pathogenic	Likely Pathogenic	Likely Pathogenic	1996/1461796	285/152278	24,6
B3GNT5	c.871A>G	p.(Asn291Asp)	VUS	NA	VUS	Likely benign	11/1461876	2/152196	23,5
VWF	c.3692A>C	p.(Asn1231Thr)	Benign	Benign	Benign	Likely benign	2632/1458198	762/152200	20,7
Sample 9									
PSEN2	c.766del	p.(Leu256Trpfs*19)	Likely pathogenic	Non presente	VUS	NA	2/1461746	0/152176	25,8
SCN5A	c.4853C>T	p.(Pro1618Leu)	VUS	VUS	VUS	NA	5/1461680	1/151828	26,8
NOTCH1	c.1838G>A	p.(Arg613His)	Benign	VUS/ 1Likely benign	VUS	Likely benign	9/1459154	0/152288	22,5
VCAM1	c.353A>T	p.(Asp118Val)	Likely benign	Non presente	VUS	Likely benign	NA	NA	NA
Sample 10									
VWF	c.974G>T	p.(Cys325Phe)	VUS	VUS	VUS	Likely pathogenic	146/1461830	155/152328	24,6
PLG	c.1748G>A	p.(Gly583Glu)	VUS	VUS	VUS	Ambiguous l_pathogenic (0.748)	16/1461750	1/152200	23,8
SH2B3	c.364G>A	p.(Glu122Lys)	VUS	NA	VUS	Likely pathogenic	1/1233240	0/151056	21,9
CLCNKB	c.968+110_1053+107del	p.(?)	NA	NA	Likely Pathogenic	NA	NA	NA	NA
SCN5A	c.2436+466_2787+17del	p.(?)	NA	NA	VUS	NA	NA	NA	NA
Sample 11									
PTCHD1	c.2396T>C	p.(Leu799Ser)	VUS	NA	VUS	Likely pathogenic	2/1097592	0/113624	25,7
CDH23	c.457G>A	p.(Val153Met)	Likely benign	VUS	VUS	Likely benign	64/1461712	3/152230	25,1
SERPINB5	c.547T>C	p.(Cys183Arg)	Likely benign	VUS	VUS	Likely benign	926/1461864	70/152314	18,3
SERPINA3	c.338C>T	p.(Ala113Val)	Likely benign	NA	VUS	Likely benign	36/1461890	6/152174	13,1
